# Continuous wave doppler pre-attenuation velocity envelope: a promising tool for the echocardiographic assessment of aortic stenosis

**DOI:** 10.1186/s44156-025-00091-2

**Published:** 2025-10-20

**Authors:** Maria Riasat, Swiri Konje, Alaa Mabrouk Salem Omar, Vikram Agarwal, Edgar Argulian

**Affiliations:** 1 Department of Cardiology, Mount Sinai Morningside, New York, NY USA; 2 Icahn School of Medicine at Mount Sinai Morningside, 1111 Amsterdam Avenue, New York, NY 10025 USA

**Keywords:** Severe aortic stenosis, LVOT measurement, Pre-attenuation jet, Spectral doppler, Echo discrepancies

## Abstract

**Background:**

In this study, we evaluated the utility of the continuous wave (CW) Doppler pre-attenuation velocity envelope as a potential surrogate for pulsed-wave (PW) Doppler-based interrogation of left ventricular outflow tract (LVOT) flow in patients with moderate or severe aortic stenosis.

**Methods:**

In a retrospective analysis, we examined 92 patients with moderate or severe aortic stenosis. Pulsed-wave Doppler was employed to acquire LVOT velocity and velocity time integral (VTI) in the 5-chamber view. CW Doppler recordings were scrutinized across multiple views with a specific focus on identifying a discernible pre-attenuation velocity envelope. Through manual tracing, we extracted peak velocity and VTI across the aortic valve as well as the pre attenuated velocity, which was used as a surrogate for LVOT assessment and substitute in the continuity equation in the evaluation of aortic valve stenosis.

**Results:**

The pre-attenuation velocity envelope was distinctly discernible in 83 (90%) of patients. PW Doppler of the LVOT velocity significantly correlated with pre-attenuation velocity from the 5-chamber view (*r* = 0.75, p-value < 0.001) but showed a weaker correlation when obtained from other windows (*r* = 0.46, p-value < 0.001). Bland-Altman analyses indicated high levels of agreement between pre-attenuation velocities from the 5-chamber view and PW Doppler derived LVOT velocities, while weaker levels of agreement were observed between pre-attenuation velocities from other windows and PW Doppler derived LVOT velocities.

**Conclusions:**

The pre-attenuation velocity envelope is attainable in the majority of patients with aortic stenosis. The pre-attenuation velocity envelope recorded from the 5-chamber view exhibits a noteworthy correlation and agreement with PW Doppler LVOT velocity. This observation positions pre-attenuation velocity envelope as a promising alternative and plausibility check for hemodynamic assessment in patients with aortic stenosis.

## Introduction

The echocardiographic determination of the aortic valve area in patients with aortic stenosis hinges on a number of measurements, each susceptible to sampling and measurement variability. Sampling left ventricular outflow tract (LVOT) velocities through pulsed wave (PW) Doppler is crucial for evaluating the severity of aortic stenosis. Accurate assessment of the LVOT flow envelope with PW Doppler poses challenges, as even slight adjustments in gate positioning, angle of recording, gain and contrast settings, as well as the location of the convergence zone can significantly impact the sampled values [1]. The guidelines suggest sampling the LVOT using PW Doppler near the aortic valve and gradually descending toward the apex until a smooth velocity curve is achieved. However, this standard method, while attempts to minimize error, does not eliminate the possibility of variability regarding sample volume positioning: slight upward motion may capture convergence zone velocities, while downward motion may underestimate true LVOT velocity. Furthermore, in the endeavor to calculate effective valve orifice area, two velocities should be sampled separately using distinct Doppler modalities, potentially amplifying temporal hemodynamic differences [2].

**Fig. 1:**
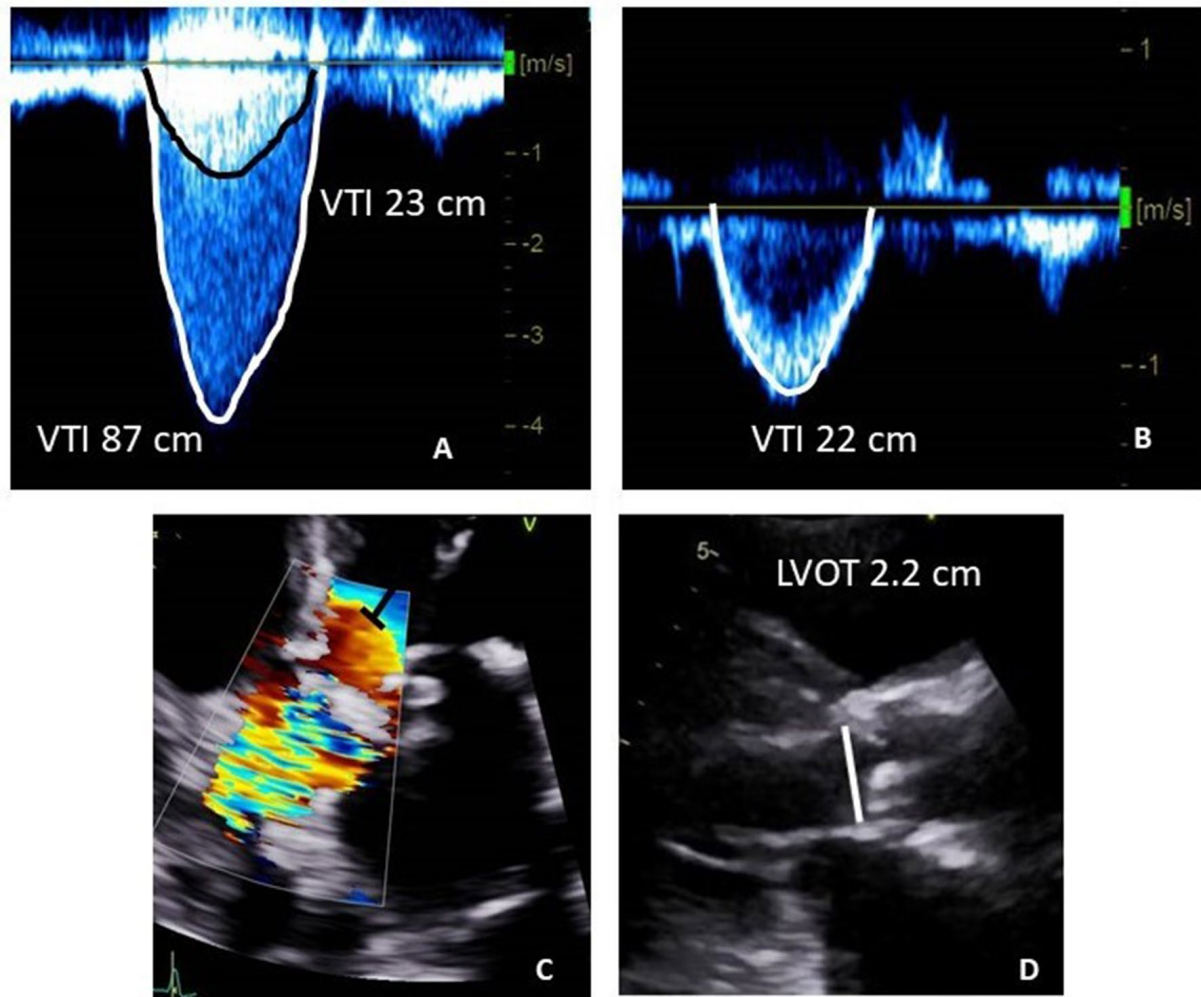
Examples of the using pre-attenuation velocity envelope for aortic valve area calculation. An example of pre-attenuation velocity envelope on CW Doppler recording (panel **A**) obtained from 5-chamber view, with clearly defined and traced envelopes. PW Doppler derived LVOT velocity from 5-chamber view is shown (panel **B**). The edge of the flow acceleration zone and recommended PW Doppler sampling area as suggested by guidelines in the 5 -chamber view is shown (panel **C**). LVOT diameter measurement in the parasternal long axis view is shown (panel **D**). Assuming the shown CW velocity is the highest obtained transaortic velocity, the aortic valve area using pre-attenuation velocity calculates at 1.00 cm2 and the aortic valve area using the standard PW Doppler calculates at 0.96 cm2

Continuous wave (CW) Doppler interrogation of the aortic valve often results in a double envelope phenomenon due to depth attenuation (Fig. 1) [2, 3]. The pre-attenuation velocity envelope aligns with the LVOT velocities, as demonstrated in prior studies using transesophageal echocardiography [4]. Our hypothesis posits that the pre-attenuation velocity envelope, observed during CW Doppler interrogation of the aortic valve, can effectively act as a surrogate for PW Doppler-based LVOT flow interrogation.

## Methods

In a retrospective study protocol, patients referred to Mount Sinai Morningside echocardiography laboratory for the assessment of moderate or severe aortic stenosis between January 2021 and September 2023 were included. Demographic, clinical, and transthoracic echocardiography data were obtained. Patients with evidence of subvalvular or supravalvular obstruction including dynamic LVOT obstruction and intracavitary gradients were excluded.

The study protocol was approved by the institutional review board.

### Echocardiographic assessments

Echocardiography was conducted using commercially available systems (Vivid E95 by GE and the Epiq CVx by Phillips), both featuring imaging and non-imaging transducers.

Assessments of the LVOT diameter and velocities across the LVOT and aortic valve were performed according to the European Association of Cardiovascular Imaging and the American Society of Echocardiography recommendations [5].

Measurement of the LVOT diameter was performed in the zoomed parasternal long axis view at the level of the aortic valve annulus, precisely from cusp insertion to cusp insertion. Gain was adjusted to optimize the blood tissue interface. The PW Doppler recordings were performed from the apical 5 chamber view, while the pre-attenuation velocity envelope was assessed in all possible windows. PW Doppler imaging was employed to obtain LVOT flow velocity. Recordings were acquired in the apical 5-chamber view with a gate size of 3–5 mm, positioned under color Doppler guidance just proximal to the region of flow acceleration, and the sample volume was moved carefully if required to obtain a laminar flow curve. The sweep speed was adjusted for optimal measurement while using low wall filter settings.

The PW recording underwent scrutiny for a narrow velocity range and was traced at the modal velocity. Manual tracing of the PW Doppler velocity envelope facilitated the extraction of LVOT peak velocity and VTI. CW Doppler was utilized to measure transaortic valvular velocities across the aortic valve from various views, including the 5-chamber view, 3-chamber views, suprasternal view, and right parasternal view (Fig. 1). Adjustments to gain, wall filters, and contrast settings were made before manual tracing to optimize envelope visualization, with the ‘invert’ function also being employed to reduce background noise and enhance the clarity of the Doppler signal. Ultrasonic enhancing agents (Perflutren lipid microsphere, Definity, Lantheus, Billerica, MA and perflutren protein-type A microspheres, Optison, GE Healthcare, Marlborough, MA) were used to enhance endocardial border definition and capture peak transaortic velocity in some patients, incorporating customized gain adjustments to mitigate blooming artifacts. However, pre-attenuation velocities were only sourced from unenhanced images. Manual tracing allowed for the extraction of peak velocity and VTI across the aortic valve, as well as the pre-attenuation velocity envelope. In patients with atrial fibrillation, we employed the maximal velocity envelope method for Doppler interrogation in estimating the aortic valve area, given its demonstrated superiority over the averaged velocity method in terms of accuracy [5–7].

### Statistical methods

Nominal data were presented as counts and percentages, with comparisons conducted using the chi-square test. Continuous data were expressed as mean ± standard deviation (SD).

To evaluate reproducibility, a subset of 10 patients was randomly selected, and pre-attenuated velocity envelopes from the 5-chamber view, were re-measured by both the same and a different operator. Intra- and inter-observer variability were assessed, reporting means ± SD of differences, along with the calculation of the interclass correlation coefficient. Correlation analyses between various methods were undertaken using univariate and multivariate linear regression, with results expressed as Pearson correlation coefficients. Agreement levels among different methods were further investigated using Bland–Altman analysis. Statistical significance was set at a p-value ⩽ 0.05. All statistical analyses were executed utilizing commercially available software (SPSS version 23.0, SPSS, Inc., Chicago, IL, USA).

### Reproducibility

In the analysis of 10 randomly selected patients, pre-attenuation velocity envelope measurements obtained from the 5-chamber view were subjected to re-measurement by both the same operator and a distinct operator. The intraobserver variability for pre-attenuation envelope peak velocity demonstrated a minimal mean difference of -0.017 ± 0.1 m/s, coupled with a robust interclass correlation coefficient of 0.86 (p-value < 0.001). Similarly, for pre-attenuation envelope VTI, the intraobserver variability yielded a mean difference of 0.5 ± 2.2 cm, with a high interclass correlation coefficient of 0.95 (p-value < 0.001).

The interobserver variability for pre-attenuation envelope peak velocity exhibited a small mean difference of 0.002 ± 0.1 m/s, accompanied by a strong interclass correlation coefficient of 0.88 (p-value < 0.001). Additionally, for pre-attenuation envelope VTI, the interobserver variability revealed a mean difference of 0.03 ± 2.1 cm, coupled with a substantial interclass correlation coefficient of 0.95 (p-value < 0.001).

## Results

A retrospective analysis was conducted on 92 consecutive patients with suspected moderate or severe aortic stenosis. However, pre-attenuation velocity envelope was not distinctly discernible in 9 patients (10%), prompting their exclusion from subsequent analyses. Consequently, the final sample size for the study comprised 83 patients, including 8 patients (10%) with atrial fibrillation.

Table 1 provides a summary of the baseline clinical and echocardiographic characteristics of the cohort. In brief, the study group exhibited a mean age of 77.5 ± 9.1 years, with 45 individuals (54%) being female. At the time of the echocardiographic assessment, the mean body mass index was 27.4 ± 6.2 kg/m2, and the systolic blood pressure averaged 133 ± 27 mmHg.

**Table 1:** Baseline characteristics of study subjects

*N* = 83	Mean ± SD
Age, years	77.5 ± 9.1
Women, n(%)	45(54)
Body mass index, kg/m^2^	27.4 ± 6.2
Hypertension, n(%)	77(93)
Diabetes mellitus, n(%)	65(78)
Hyperlipidemia, n(%)	75(90)
Systolic blood pressure, mmHg	133 ± 27
Diastolic blood pressure, mmHg	68 ± 11.3
Windows with pre-attenuation envelope, n(%)	
5ch	83(100)
3ch	71(86)
Non imaging probe	54(65)
Peak pulsed-wave LVOT velocity, m/s	0.93 ± 0.18
Pulsed-wave LVOT VTI, cm	20.9 ± 5.4
Peak velocity of pre-attenuated envelope in 5-chamber view, m/s	0.96 ± 0.17
VTI of pre-attenuated envelope in 5-chamber view, cm	20.1 ± 5.1
Peak velocity of pre-attenuated envelope in other windows, m/s	0.94 ± 0.21
VTI of pre-attenuated envelope in other windows, cm	19.6 ± 7.2
LVOT diameter, cm	2.08 ± 0.17
Aortic valve VTI by continuous wave, cm	72.3 ± 24.8
Aortic valve area, cm^2^	1.06 ± 0.26
≥ 1/<1 cm^2^	51(61)/32(39)
Left ventricular ejection fraction, %	52.1 ± 15.5
Left ventricular mass index, gm/m^2^	116.2 ± 57.6

LVOT left ventricular outflow tract, VTI velocity time integral

### Echocardiographic parameters

Within the study population, the mean ejection fraction was 52 ± 16% [< 50% in 25(30%) patients], and the left ventricular mass index was 116.2 ± 57.6 gm/m2. The mean LVOT diameter measured 2.1 ± 0.17 cm, while the PW Doppler LVOT peak velocity was recorded at 0.94 ± 0.21 m/s, and the VTI was 21 ± 5.4 cm. Furthermore, the mean peak transaortic velocity was 3.2 ± 0.7 and mean gradient was 26.4 ± 13.5 mmHg, with a VTI of 72 ± 24.8 cm, and the calculated mean aortic valve area was 1.06 ± 0.26 cm2. Notably, 11 (13%) patients presented with significant aortic regurgitation, encompassing 2 cases of severe and 9 of moderate severity. In contrast, 72 (87%) patients exhibited no more than mild aortic regurgitation. As indicated by the aortic valve area, 32 patients (39%) were identified with severe aortic stenosis (≤ 1.0 cm2), while 51 patients (61%) demonstrated non-severe aortic stenosis (AVA > 1.0 cm2).

### Pre-attenuation velocity envelope

**Fig. 2:**
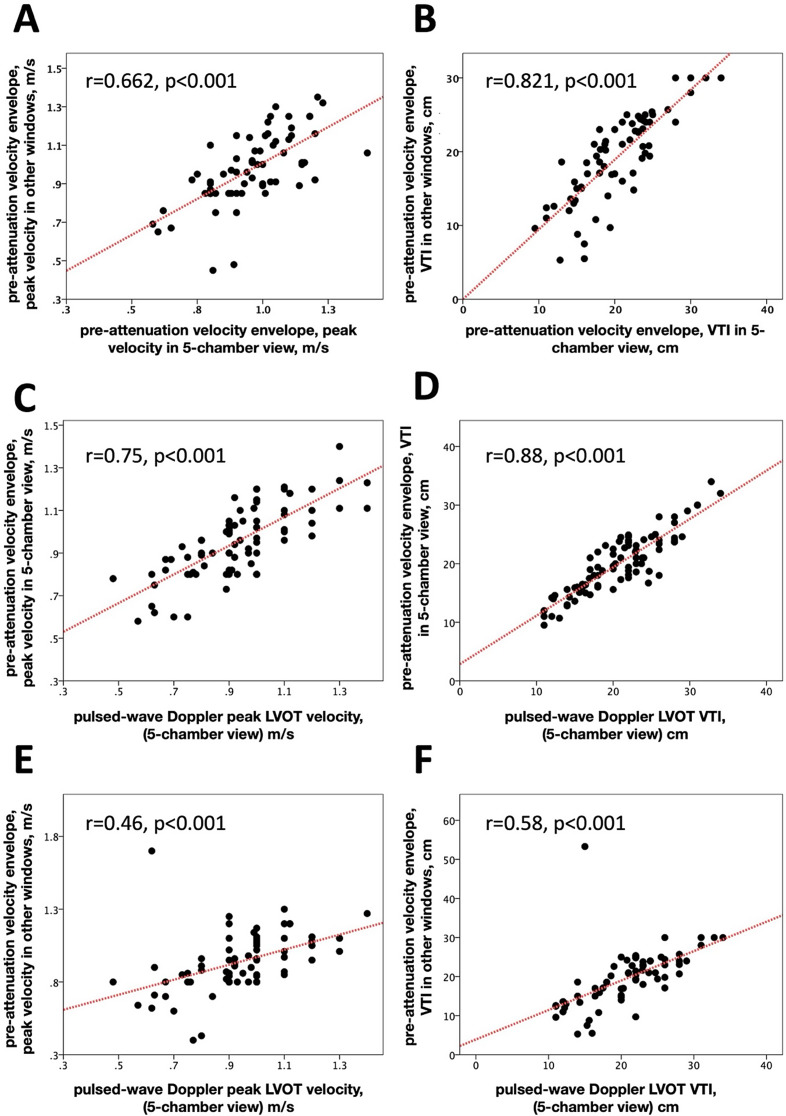
Dots plots showing Pearson correlations. **A**, **B** Correlations between velocities from pre-attenuated velocity envelope in the 5-chamber view and other windows. **C**, **D**, Correlations between velocities from pre-attenuated velocity envelope in the 5-chamber view and PW Doppler derived LVOT velocities. **E**, **F**, Correlations between velocities from pre-attenuation velocity envelope in other windows and pulsed-wave Doppler derived left ventricular outflow tract velocities. LVOT (left ventricular outflow tract), VTI (velocity time integral)

The pre-attenuation velocity envelope was consistently observed in all patients through CW Doppler recordings from the apical 5-chamber view, and it was successfully obtained in 71 (86%) patients from the 3-chamber view. Additionally, pre-attenuated velocity was recorded using a non-imaging probe in 54 (65%) patients. The highest velocity values for pre-attenuated signals were derived from the 5-chamber view in 55 (66%) patients, the 3-chamber view in 16 (19%) patients, and the non-imaging probe in 12 (14%) patients. In the context of the 5-chamber view, the mean pre-attenuated peak velocity was measured at 0.96 ± 0.17 m/s, with a mean VTI of 20.1 ± 5.1 cm. Comparatively, when the pre-attenuated envelope was obtained from an alternative window, the peak velocity recorded was 0.94 ± 0.2 m/s, accompanied by a mean VTI of 19.6 ± 7.2 cm. Analyzing the relationship between peak pre-attenuated velocity obtained from the 5-chamber view and that obtained from another window revealed a moderate correlation (*r* = 0.662, p-value < 0.001, Fig. 2A). Furthermore, the pre-attenuated VTI obtained from the 5-chamber view exhibited a robust correlation with VTI obtained from other windows (*r* = 0.821, p-value < 0.001, Fig. 2B).

### Correlation between PW doppler and pre-attenuation velocity envelope

**Fig. 3:**
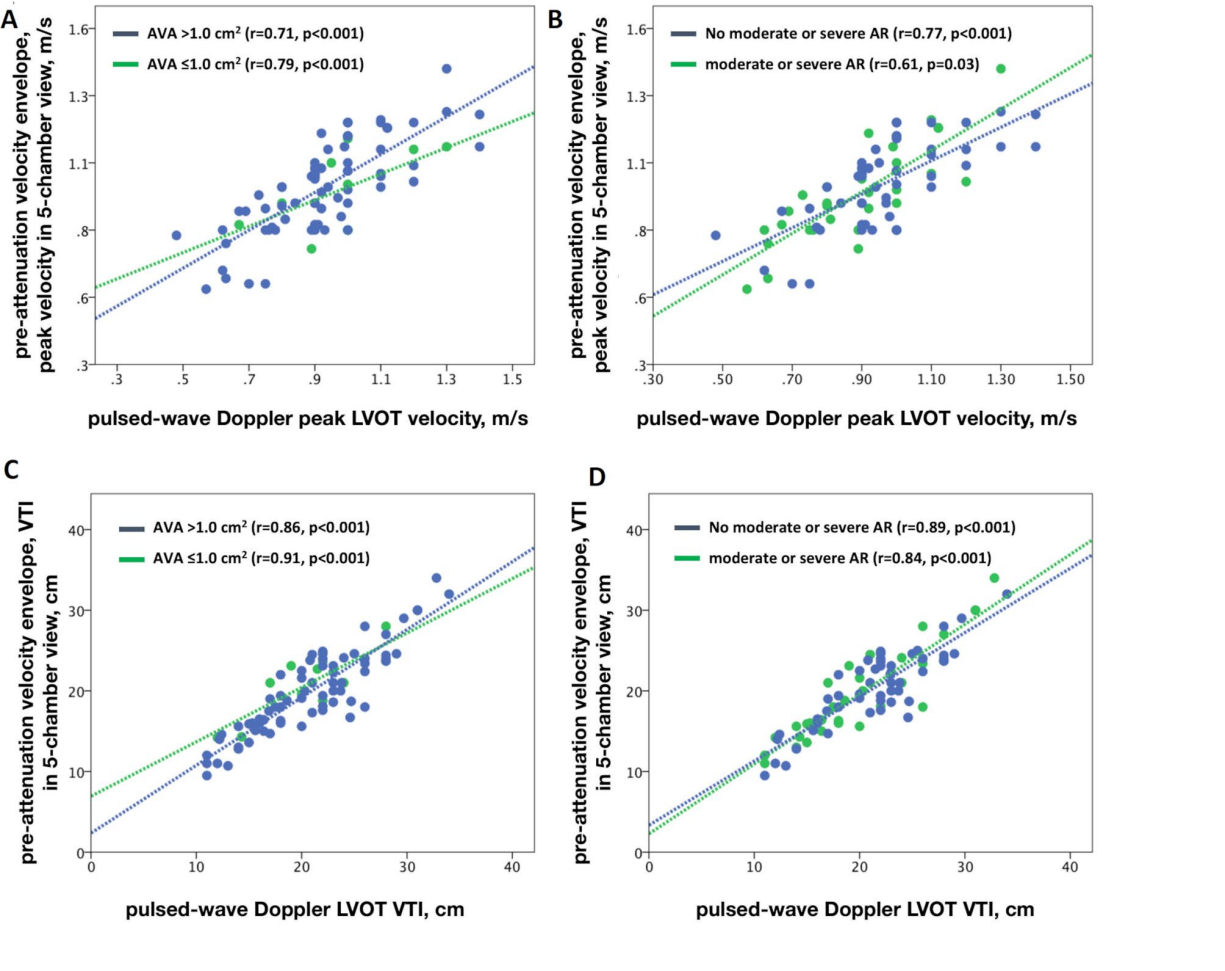
Dots plots showing Pearson correlations in subgroups. **A**, **B**, Correlations between peak velocities from pre-attenuation velocity envelope in the 5-chamber view and pulsed-wave Doppler derived left ventricular outflow tract velocities in subgroups classified based on aortic valve area (AVA, panel **A**) and the presence or absence of significant aortic regurgitation (AR, panel **B**). **C**,**D**, Correlations between velocity time integral derived from pre-attenuation velocity envelope in the 5-chamber view and pulsed-wave Doppler derived left ventricular outflow tract velocities in subgroups classified based on AVA (panel **C**) and the presence or absence of significant AR (panel **D**). LVOT (left ventricular outflow tract), VTI (velocity time integral)

PW Doppler LVOT velocity exhibited a significant correlation with pre-attenuation envelope peak velocity when obtained from the 5-chamber view (*r* = 0.75, p-value < 0.001, Fig. 2C), as well as when acquired from alternative windows (*r* = 0.46, p-value < 0.001, Fig. 2E). Multivariate analysis revealed that only pre-attenuation envelope peak velocity obtained from the 5-chamber view maintained a correlation with PW Doppler LVOT peak velocity (Beta = 0.71, p-value < 0.001, Table 2), while the correlation was lost for pre-attenuation envelope peak velocity obtained from other windows (Beta = 0.09, p-value = 0.402, Table 2). Similarly, PW VTI exhibited a significant correlation with pre-attenuation envelope VTI when obtained from the 5-chamber view (*r* = 0.88, p-value < 0.001, Fig. 2D) and when acquired from other windows (*r* = 0.58, p-value < 0.001, Fig. 2F). Multivariate analysis indicated that only pre-attenuation envelope VTI obtained from the 5-chamber view correlated with PW Doppler LVOT VTI (Beta = 0.85, p-value < 0.001, Table 2), while the correlation was lost for pre-attenuation envelope VTI obtained from other windows (Beta = 0.07, p-value = 0.198, Table 2). Crucially, the robust correlation observed between pre-attenuation envelope velocity measurements from the 5-chamber view and PW Doppler LVOT velocity measurements persisted across subgroups stratified by the presence or absence of significant aortic regurgitation and aortic valve area categories (≤ 1 cm2 and > 1 cm2, Fig. 3).

**Table 2:** Univariate and multivariate correlations of outflow tract velocities obtained using pre-attenuation envelope with pulsed-wave doppler

	Univariate		Multivariate	
	r	p-value	Beta	p-value
Maximum velocity				
Pre-attenuation from 5 chamber	0.75	< 0.001	0.71	< 0.001
Pre-attenuation from alternative windows	0.46	< 0.001	0.09	0.402
Velocity time integral				
Pre-attenuation from 5 chamber	0.88	< 0.001	0.85	< 0.001
Pre-attenuation from alternative windows	0.58	< 0.001	0.07	0.198

### Agreement between PW doppler and pre-attenuation velocity envelope

**Fig. 4:**
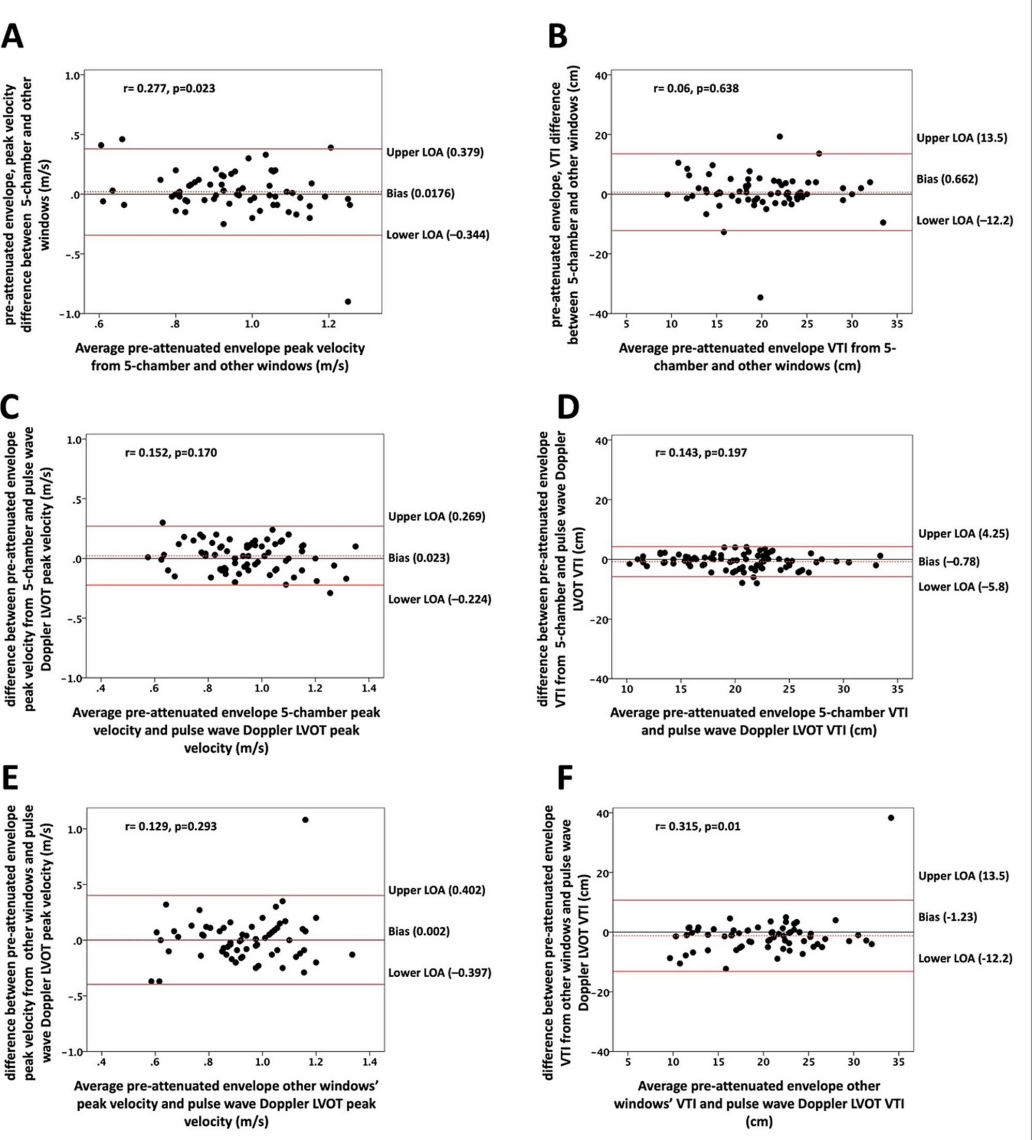
Bland Altman plots for assessment of agreement between different methods. **A**, **B** Agreement between pre-attenuation envelope peak velocity (panel **A**) and VTI (panel **B**) in the 5-chamber view and other windows. **C**, **D**, Agreement between pre-attenuation envelope peak velocity (panel **C**) and velocity time integral (panel **D**) in the 5-chamber view and PW Doppler derived LVOT velocities. **E**, **F**, Agreement between pre-attenuation peak velocity (panel **E**) and VTI (panel **F**) in other windows and PW Doppler derived LVOT velocities. LVOT (left ventricular outflow tract), VTI (velocity time integral)

The differences between pre-attenuation envelope peak velocity obtained in the apical 5-chamber view and other windows were small, with a mean difference of 0.02 ± 0.18 m/s, and for VTI, the mean difference was 0.66 ± 6.6 cm. Bland-Altman analysis revealed a modest level of agreement, ranging from 0.379 to -0.344 m/s for peak velocity (Fig. 4A) and 13.5 to -12.2 for VTI (Fig. 4B), indicating only moderate consistency between measurements from the 5-chamber view and other windows. Furthermore, when comparing pre-attenuation envelope peak velocity obtained from the apical 5-chamber view with PW Doppler-derived peak LVOT velocity, the mean difference was 0.023 ± 0.16 m/s, and for VTI, it was − 0.78 ± 2.6 cm. Conversely, the differences between pre-attenuation envelope peak velocity obtained from other windows and PW Doppler-derived peak LVOT velocity were 0.002 ± 0.2 m/s, and for VTI, they were − 1.27 ± 6.1 cm. Bland-Altman analysis illustrated a high level of agreement between pre-attenuation envelope velocities from the 5-chamber view and PW Doppler-derived peak LVOT velocity, ranging from 0.269 to -0.224 m/s for peak velocity (Fig. 4C) and 4.25 to -5.8 for VTI (Fig. 4D). Conversely, the agreement between pre-attenuation envelope velocities from other windows and PW Doppler-derived peak LVOT velocity velocities was weaker, with levels ranging from 0.402 to -0.397 m/s for peak velocity (Fig. 4E) and 13.5 to -12.2 for VTI (Fig. 4F), compared to the robust agreement observed for pre-attenuation envelope velocities derived from the 5-chamber views.

## Discussion

Doppler echocardiography plays a crucial role in evaluating the severity and hemodynamics of aortic stenosis [5]. The continuity equation and the dimensionless index are well-known Doppler-dependent methods used in aortic stenosis assessments. However, challenges in obtaining a satisfactory LVOT velocity can significantly bias Doppler assessments [6]. The current study confirms that the transaortic CW Doppler tracing often exhibits a second lower-velocity pre-attenuation velocity waveform believed to reflect LVOT velocity. The findings suggest that this velocity can be obtained from multiple views including tracing obtained using non-imaging probes. However, our findings suggest that the pre-attenuation velocity envelope demonstrates the strongest correlation and excellent agreement with PW Doppler–derived LVOT velocity when acquired from the apical 5-chamber view. The current study proposes that the pre-attenuation velocity envelope closely aligns with conventionally measured LVOT velocity by PW Doppler and could serve as a viable alternative.

The American Society of Echocardiography endorses the utilization of PW recording as the standard method for the acquisition of the LVOT Doppler signal. This recommendation stems from theoretical concerns regarding the impact of higher velocities within the flow convergence zone proximal to the stenosis on the pre-attenuation signal accuracy [6]. Interestingly, the current study, as well as preceding research, have failed to validate these concerns. Both the present investigation and past studies have consistently demonstrated robust agreement between the pre-attenuation envelope method and the PW Doppler method [7]. Moreover, there is no signal in these studies that suggests overestimation of the aortic valve area by the pre-attenuation method.

The pre-attenuation velocity pattern has been previously described as a result of the abrupt acceleration of left ventricular outflow tract (LVOT) flow toward the stenotic aortic valve, producing an initial dense Doppler signal. This is followed by a less dense envelope leading up to the peak velocity, which corresponds to the maximum velocity across the stenotic valve orifice [8].

This alternative method presents several advantages over PW Doppler velocities. The standard PW Doppler approach introduces subjectivity related to sample volume placement and alignment of the interrogation angle. Moreover, it often captures cardiac cycles that are not temporally aligned with those used to obtain CW Doppler–derived transaortic velocities, potentially leading to inconsistencies in derived hemodynamic measurements. Importantly, in situations involving fast and irregular heart rates or arrhythmias, careful consideration is essential when measuring LVOT velocities. While averaging measurements over multiple cardiac cycles can enhance reliability, it introduces complexity and introduces an inevitable source of error. Using the pre-attenuation velocity may overcome these limitations.

Firstly, the pre-attenuation velocity naturally produces a second velocity envelope without requiring observer intervention, mitigating operator-dependent corrections associated with false high velocities due to the convergence zone at the level of the stenotic valve. Secondly, using the pre-attenuation velocity envelope in a single view for both aortic valve and outflow tract measurements minimizes temporal differences, beat-to-beat variability, and ensures that angle inaccuracies and changes in hemodynamics affect outflow tract and transaortic valve velocities uniformly. Therefore, the use of pre-attenuation velocity appears theoretically more favorable for assessing aortic stenosis via Doppler echocardiography. This method simplifies the assessment process as well as offers plausibility check mechanism for values obtained by conventional PW Doppler.

The current study has several important limitations. First, the sample size was small, limiting the statistical power and generalizability of the findings; further validation in larger, prospective cohorts is warranted. Acceptable correlation and agreement were observed for pre-attenuation envelope velocity measurements obtained from the apical 5-chamber view, while weaker correlations were noted in other imaging windows. The need for manual adjustments of gain and contrast settings to delineate envelope contours introduces a degree of subjectivity to the measurements and may limit reproducibility. Importantly, the pre-attenuation velocity approach does not overcome the fundamental challenge of achieving optimal alignment between the Doppler beam and blood flow. The strongest correlation was noted when using the apical 5-chamber view, likely due to its favorable anatomic alignment with LVOT flow. In this study, LVOT velocities were measured exclusively from the apical 5-chamber view. Current guidelines recommend assessment from either the apical 5-chamber or 3-chamber views. It remains uncertain whether using the apical 3-chamber view would have produced different results or altered severity classification.

Reproducibility analyses were conducted in a limited subgroup of 10 patients, precluding subgroup comparisons. A small number of patients were excluded due to the absence of a clearly defined pre-attenuation velocity envelope. No further analysis was performed on this group, and the underlying reasons for poor envelope definition remain unclear. Future studies should further investigate the mechanisms behind the absence of a clear pre-attenuation envelope.

Finally, the pre-attenuation velocity approach does not address the limitations of Doppler assessment in patients with serial LVOT obstruction, which remains a diagnostic challenge.

In summary, the current study demonstrates the feasibility of capturing the pre-attenuation velocity signal in the majority of patients with aortic stenosis. Notably, the pre-attenuation velocity recorded from the 5-chamber view exhibits a significant correlation with excellent levels of agreement with PW Doppler LVOT velocity.

## Data Availability

No datasets were generated or analysed during the current study.
